# Establishment of cell lines with increased susceptibility to EV71/CA16 by stable overexpression of SCARB2

**DOI:** 10.1186/1743-422X-10-250

**Published:** 2013-08-06

**Authors:** Xiaojun Li, Peihun Fan, Jun Jin, Weiheng Su, Dong An, Lin Xu, Shiyang Sun, Yan Zhang, Xiangyu Meng, Feng Gao, Wei Kong, Chunlai Jiang

**Affiliations:** 1 School of Life Sciences, Jilin University, Changchun, P.R. China; 2 National Engineering Laboratory for AIDS Vaccine, Jilin University, Changchun, P.R. China; 3 Key Laboratory for Molecular Enzymology & Engineering, the Ministry of Education, Jilin University, 2699 Qianjin Street, Changchun 130012, P.R. China; 4 Human Vaccine Institute, Duke University Medical Centre, Durham, NC 27710, USA

**Keywords:** SCARB2, EV71/CA16, 293 cells, RD cells, Vero cells

## Abstract

**Background:**

Human enterovirus type 71 (EV71) and Coxsackievirus A group type 16 (CA16) belong to human *Enterovirus* species A of the family *Picornaviridae*. These viruses are recognized as the major pathogens responsible for epidemics of hand-foot-mouth disease (HFMD), which presents with fever and vesicular eruptions of palms, soles of the feet or mouth. Human scavenger receptor class B, member 2 (SCARB2) has been identified as the receptor for both EV71 and CA16, as overexpression of SCARB2 in cells can enhance virus replication significantly.

**Methods:**

In this study, we used a lentivirus packaging vector to transduce the *SCARB2* gene into human embryonic kidney cells (293), human rhabdomyosarcoma cells (RD) and African green monkey kidney cells (Vero) to create stable expression lines. Expression of SCARB2 in the resulting three transgenic cell lines was confirmed by real-time RT-PCR, immunofluorescence and flow cytometry.

**Results:**

Levels of *SCARB2* mRNA determined by real-time RT-PCR in 293-SCARB2 (293S) or RD-SCARB2 (RDS) transgenic cell lines were approximately 2 × 10^2^ times higher than those in 293 and RD cells, respectively, and three times higher in Vero-SCARB2 (VeroS) than in Vero cells. Furthermore, EV71 and CA16 virus titers in 293S and RDS cells were 10^2^–10^3^-fold higher (detected in RD cell) than those in the parental cells, and a 10-fold higher titer of EV71 was achieved in VeroS cells compared with that in Vero cells.

**Conclusions:**

We established for the first time three cell lines stably overexpressing SCARB2, which showed drastic increases in susceptibility to EV71/CA16 infection. These optimal cell lines may be utilized to develop inactivated vaccines for EV71/CA16 and facilitate rapid detection and isolation of HFMD pathogens or other *Enterovirus* serotypes. Furthermore, these stable cell lines also can serve as tools to facilitate drug screenings as well as molecular studies on virus-host interactions and pathogenesis of causative agents for HFMD.

## Background

EV71 and CA16, both belonging to human *Enterovirus* species A of the *Enterovirus* genus within the family *Picornaviridae*, are recognized as major pathogens responsible for hand-foot-mouth disease (HFMD) epidemics [1,2]. Although HFMD generally produces mild exanthems, infections involving EV71 can progress to severe neurological disease, including fatal encephalitis, aseptic meningitis and acute flaccid paralysis [3-7]. Neurological complications of EV71 infection also may occasionally cause permanent paralysis or death. The first case of HFMD was reported in the United States in 1969 [8]. Subsequent epidemics and sporadic outbreaks of HFMD have been reported in China, Malaysia and many other countries, especially in Asia [9]. Thus, HFMD has become a considerable public health concern worldwide.

Effective antiviral agents and vaccines against EV71 or CA16 are currently unavailable [10,11]. In addition, rapid and low-cost diagnostic methods for identifying infections of pathogens causing HFMD, especially for EV71, are also needed [12,13]. Virus isolation and conventional assays, such as neutralization assays and RT-PCR, for viral RNA detection and diagnosis of EV71/CA16 are time-consuming and labor-intensive [14-16]. Although cell culture is most widely used in laboratory diagnosis and to identify different serotypes in preclinical studies, there are no optimal cells lines which are susceptible to the major pathogens of HFMD. Therefore, choosing or establishing an ideal cell line is essential for improving virus isolation and propagation methods for vaccine development.

Recently, an inactivated EV71 vaccine made from Vero or KMB-17 cells was developed [17-20]; however, the virus titer did not reach high levels in these cell lines. Specific cell surface receptors are important for permitting infection of many viruses. Two membrane proteins have been identified as cellular receptors of EV71 and CA16, human P-selectin glycoprotein ligand-1 (PSGL-1) [21] and human scavenger receptor class B, member 2 (SCARB2) [22]. SCARB2, also called lysosomal integral membrane protein 2 (LIMP-2), lysosomal membrane glycoprotein-85 (LGP85) or CD36b like-2, has two transmembrane domains with the N and C termini located in the cytosol and traverses the membrane twice. Expressed in many tissues, SCARB2 is proposed to be the receptor for the selective uptake, degradation of cytosolic proteins by lysosomes [23] and endosomal/lysosomal compartments [24]. In addition, overexpression of SCARB2 in L929 cells has been demonstrated to increase the titer of EV71/CA16 strains [22,25-27].

Furthermore, previous reports have shown that transient overexpression of SCARB2 in cells which naturally lack or express low levels of this receptor can yield higher virus titers of EV71 and CA16 [22]. RD and Vero cell lines have been most widely used for culture of most enteroviruses [12,18], and CA16 virus titers in 293 cells are generally higher than those in other cells, such as Vero and A549 cells (previously observed in our lab). Therefore, in this study, *SCARB2* gene was introduced into 293, RD and Vero cells separately via a lentiviral expression vector and the susceptibility of stable SCARB2-overexpressing cells to infection by EV71 and CA16 would be significantly enhanced compared with the parental cells.

## Results

### Establishment of cell lines stably expressing SCARB2

To establish cell lines stably expressing a high level of SCARB2, the 293, RD and Vero cell lines were transduced with a lentivirus carrying the *SCARB2* gene, and lentivirus production was detected in the supernatant. Positive colonies were selected in the presence of puromycin and sub-cloned three times. After selection, at least 10 puromycin-resistant cell colonies were screened, and two clones expressing the highest levels of SCARB2 were selected for subsequent experiments (data of one clone shown). Compared with the parental cells, SCARB2 expression in the cell lines detected every five passages showed a higher SCARB2 expression by real-time RT-PCR and flow cytometry (data not shown). Furthermore, the size and form of the stable SCARB2-expressing cells appeared similar to those of the original cell lines, except for RDS cells which exhibited a plumper polygonal cell morphology compared with RD cells (data not shown).

### Analysis of stable cell lines

**Figure 1:**
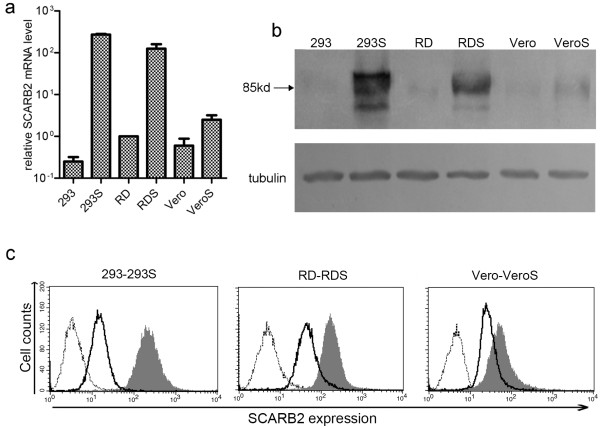
**Detection of SCARB2 expression in SCARB2-overexpressing and parental cells. (a)** Relative *SCARB2* mRNA level was detected by real-time RT-PCR with *β-actin* as the internal control. **(b)** SCARB2 protein of six cells was detected by Western blot using an anti-SCARB2 antibody. **(c)** Cell surface expression of SCARB2 in six cells. Three parental cells were stained with an anti-SCARB2 antibody (solid lines), SCARB2 cells were stained with an anti-SCARB2 antibody (grey region) or a secondary antibody alone (dotted lines) and analyzed by flow cytometry.

Real-time RT-PCR was used to examine the relative expression of *SCARB2* transcripts. The transgenic cell lines were able to stably express up to 2 × 10^2^-fold higher levels of the *SCARB2* mRNA compared to the original cell lines (Figure 1a). Western blot analysis using an anti-SCARB2 antibody indicated that SCARB2 protein levels in 293S, RDS and VeroS cells were obviously higher than those expressed at basal levels in the parental cells (Figure 1b). Among the three stable cell lines, 293S and RDS evidently expressed the highest amounts of SCARB2 at both the transcriptional and protein levels, which was confirmed concurrently by flow cytometry analysis (Figure 1c). Altogether, these results indicated that the 293S, RDS and VeroS cells stably expressed SCARB2 on the cell surface after screening and selection, with 293S and RDS showing the highest levels of the receptor.

### Localization of SCARB2

**Figure 2:**
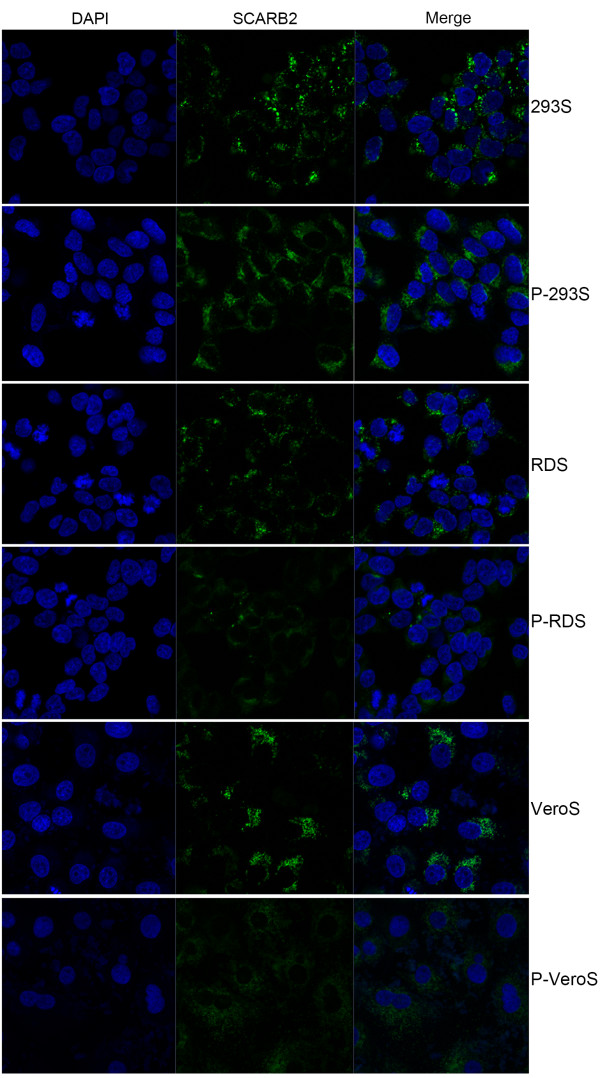
**Localization of SCARB2.** Cells were fixed and stained with a SCARB2-specific antibody (green) at a suboptimal concentration that did not detect endogenous SCARB2 proteins in the cell membrane of the three parental cell lines. Nuclei were stained with DAPI (blue). The three stable cell lines were treated with (P-cell) or without Triton-100 to permeabilize the cell membrane.

To determine the localization of SCARB2 in 293S, RDS and VeroS cells, we monitored the receptor expression by confocal microscopy. Cell surface expression of SCARB2 was clearly observed on all three transgenic cells. Every cell line was permeabilized (P-cell) by Triton-100 or not and stained using a suboptimal concentration of antibody that did not stain the endogenous SCARB2 on the cell membrane of the three parental cell lines. As shown in Figure 2, SCARB2 was more dispersed in the cytoplasm of cells treated with Triton-100, while SCARB2 was observed at the cell membrane when the cells were not permeabilized. These data confirmed that SCARB2 was localized to the surface membrane in the three transgenic cell lines.

### Characterization of SCARB2 stable cell lines

**Figure 3:**
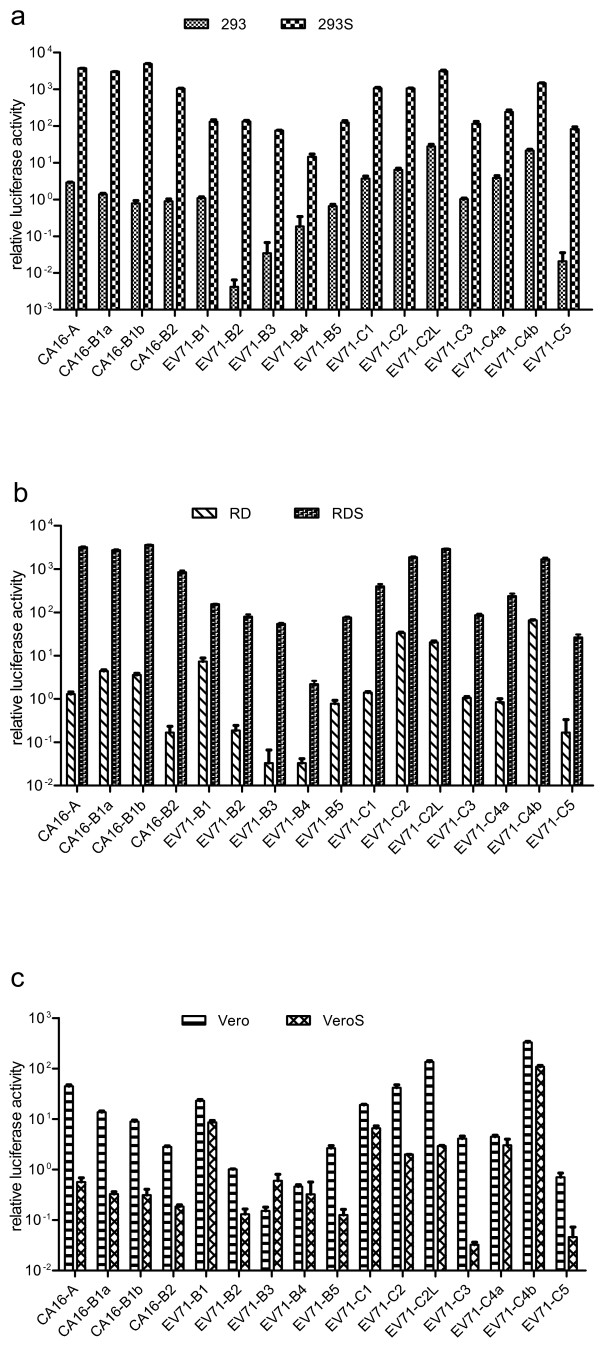
**Infectivity of EV71 and CA16 pseudoviruses in SCARB2-overexpressing and parental cells.** Twelve serotypes of EV71 pseudoviruses and four serotypes of CA16 pseudoviruses with the luciferase reporter gene were used to infect 293 and 293S cells **(a)**, RD and RDS cells **(b)**, Vero and VeroS cells **(c)**.

**Figure 4:**
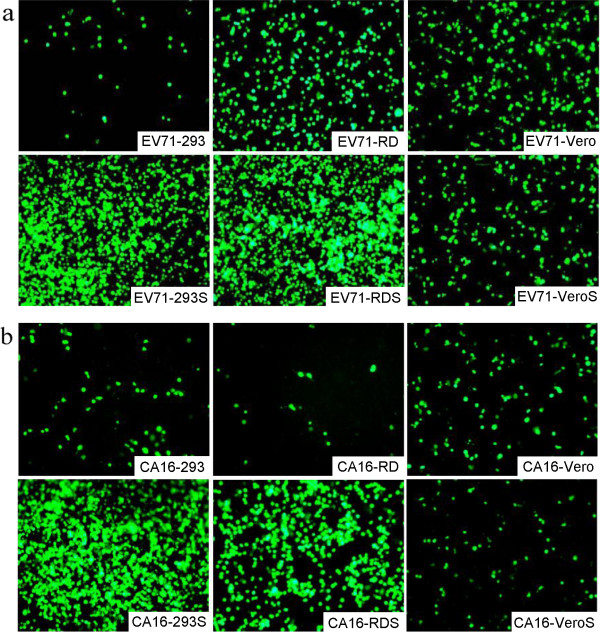
**Detection of EV71-EGFP and CA16-EGFP virus in infected cells.** The six cells were infected with the EV71-EGFP **(a)** or CA16-EGFP **(b)** virus, and images were acquired using a fluorescence microscope after incubation for 48 h.

Infectibility of the stable transgenic cell lines was compared with that of the original cells using EV71 and CA16 pseudoviruses and wild-type viruses. First, twelve genotypes of EV71 pseudovirus and four genotypes of CA16 pseudovirus carrying the luciferase reporter gene were used to infect the six cell lines. Luciferase measurements indicated that all of the pseudoviruses produced higher titers in the SCARB2 cell lines than in the parental cells (Figure 3). Almost all EV71 and CA16 pseudoviruses showed 10^2^–10^4^-fold higher titers in 293S and RDS cells than those in the parental cells, while no significant differences in titers, except for the slightly higher level of EV71-B3, were observed in VeroS versus Vero cells. Concurrently, the six cell lines were infected with EV71 and CA16 viruses expressing the EGFP reporter protein. Subsequent analysis showed higher levels of EGFP protein in 293S and RDS cells than in 293 and RD cells, respectively. However, no significant difference in the level of EV71-EGFP or CA16-EGFP was found between infected VeroS and Vero cells (Figure 4). These results demonstrated that 293S and RDS were more sensitive than parental cells to infection of EV71 or CA16.

### Replication kinetics

**Figure 5:**
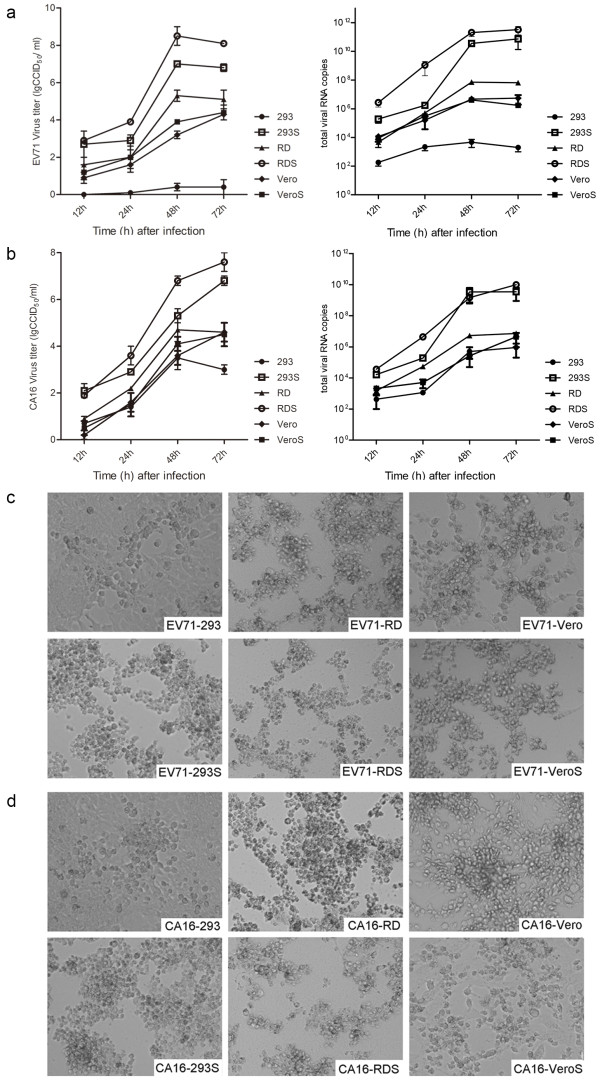
**Viral growth kinetics in SCARB2-overexpressing and parental cells.** Viral growth kinetics of EV71 **(a)** and CA16 **(b)** were detected by determination of CCID_50_ in RD cells and real-time RT-PCR. **(c, d)** Images of CPE were acquired at 48 h after wild-type EV71 and CA16 infection in the six cell lines.

To assess the efficiency of virus infection in SCARB2-overexpressing and parental cells, EV71 and CA16 wild-type viruses were used to infect the six cell lines, which were harvested at 12, 24, 48 and 72 h after infection. The growth kinetics of EV71 and CA16 were determined by titration in RD cells (CCID_50_) and real-time RT-PCR. As shown in Figure 5a and 5b, EV71 and CA16 virus titers in 293S and RDS cells were 10^2^–10^3^-fold higher than those in the parental cells, and a 10-fold higher titer of EV71 was achieved in VeroS cells compared with that in Vero cells. Meanwhile, the CCID_50_ values of EV71 and CA16 virus collected at 24 and 48 h post-infection (hpi) from these six cell lines were dramatically elevated in both 293S and RDS, consistent with the real-time RT-PCR results. However, EV71 was reduced as determined by CCID_50_ titer but not by real-time RT-PCR at 72 hpi, while CA16 was still elevated by both measures at that time. Additionally, observations at 48 h after EV71 and CA16 infection in the six cell lines showed that the CPE in the transgenic cells initiated earlier and increased more evidently and rapidly than in parental cells (Figure 5c and 5d). As with the results above, there were still no significant differences in CCID_50_ and viral mRNA of CA16 between Vero and VeroS cells. These results indicated that EV71 and CA16 could enter and propagate in the SCARB2 transgenic cells and spread efficiently and rapidly.

## Discussion

As major causative pathogens of HFMD epidemics, EV71 and CA16 can lead to severe neurological disease. No effective antiviral agents or vaccines against EV71 and CA16 or rapid and low-cost diagnostic methods for these viruses are currently available. Conventional detection and isolation methods for HFMD pathogens from clinical samples involve co-culture with cell lines such as RD, A549 and BGMK cells, followed by RT-PCR or CPE examination. Rapid detection of pathogens depends on the selection of cell lines, and RD cells had been the most sensitive cell type in previous studies [12,28-32]. Although the titers were not high, an inactivated EV71 vaccine has been generated from Vero or KMB-17 cells [17-20]. The Vero cell line is also the most widely accepted continuous cell line by regulatory authorities and has been used for the production of polio and H5N1 inactivated whole-virus vaccines [33]. Meanwhile, high CA16 virus titers have been achieved with 293 cells (previously observed in our lab).

Based on the reasons above, we selected the 293, RD and Vero cell lines to prepare transgenic cell lines expressing SCARB2, one of the known receptors of HFMD pathogens that are widely expressed in different cells and tissues. As the overexpression of SCARB2 in L929 has been shown to increase EV71 binding to cells and yield higher virus titers [22], we reasoned that SCARB2 expression in 293, RD and Vero cells should also facilitate infectivity and propagation of HFMD pathogens. The transgenic cell lines were generated using a lentivirus-based vector, a method which is widely used for inserting a gene of interest into cells or animals for research or into organs for human transplantation [34-37]. In this study, we used the Lenti-X Plvx-puro vector for packaging lentiviruses with the VSV-G envelope, which allows transduction of almost all mammalian cells [38], to generate the SCARB2 stable cell lines. Monitoring of the transduced cell lines by real-time RT-PCR and flow cytometry every five passages showed that SCARB2 was stably overexpressed for up to 50 passages (data not shown). 293S and RDS showed the greatest increases of *SCARB2* mRNA levels (>200-fold) and titers of EV71 and CA16 (>10^3^-fold), compared with parental cells. EV71 achieved an especially high titer in 293S that was 10^7^-fold higher than that in 293 cells. Meanwhile, VeroS cells produced only 10-fold higher virus titers compared with Vero cells, which was consistent with its lower expression of SCARB2 relative to that of 293S or RDS cells.

Some distinguishing characteristics were observed between the propagation of EV71 and CA16 in the stable cell lines. First, the EV71 virus titer was 10 ~ 100-fold higher than that of CA16 when used to infect 293S or RDS cells separately, and this difference was 10-fold in VeroS cells. These results imply that EV71 is more sensitive to SCARB2 and better utilizes this protein receptor than CA16. However, there was a dramatic 10^7^-fold elevation in the titer of wild-type EV71 virus (EV71-C4b), while the EV71 pseudovirus (also EV71-C4b) generated a 10^3^-fold higher titer in 293S cells than in 293 cells. The lower increase in titer seen with the EV71 pseudovirus could be explained by the fact that it undergoes a single round of infection, while the wild-type virus replicates multiple times before detection.

Examination of viral growth kinetics by measuring CCID_50_ revealed that virus attachment in the first step of infection (primary infection) and replication of EV71 and CA16 were more efficient when cells overexpressed the SCARB2 protein. More obvious differences of viral mRNA were detected than those found by observing CPE in RDS cells due to the greater sensitivity of real-time RT-PCR. However, viral replication was affected in cells displaying CPE over a long period of time (beyond 12–24 hpi), and there was a mild decrease in EV71 titer at 72 hpi. This phenomenon is similar to that reported by Yamayoshi *et al*. [22]. Taken together, the results suggest that these stable cell lines may support increased rates of infection and replication of EV71 and CA16.

In summary, we successfully established three transgenic cell lines using a lentivirus system to stably overexpress the human SCARB2 protein on the surface of 293, RD and Vero cells. The SCARB2-overexpressing cells will facilitate more rapid detection and isolation of the major pathogens of HFMD, as well as other serotypes of enteroviruses such as CA7 and CA14 which share the SCARB2 receptor [26].

## Conclusion

In this study, we established for the first time three cell lines with increased susceptibility to EV71/CA16 by stable overexpression of SCARB2, in which EV71 and CA16 virus titers were 10^2^–10^3^-fold higher than those in the parental cells.

## Materials and methods

### Cells

Human embryonic kidney (HEK 293), human rhabdomyosarcoma (RD), African green monkey kidney (Vero) and 293T cells were obtained from the American Type Culture Collection (ATCC) and maintained in Dulbecco’s modified Eagle medium (DMEM) (Sigma) supplemented with 10% fetal bovine serum (FBS) (10% FBS-DMEM). Cells were maintained at 37°C with 5% CO_2_. The stable cell lines 293-SCARB2 (293S), RD-SCARB2 (RDS) and Vero-SCARB2 (VeroS) were cultured in 10% FBS-DMEM supplemented with puromycin (293S: 1.25 μg/ml, RDS: 0.5 μg/ml, VeroS: 4.0 μg/ml; Clontech).

### Viruses

#### Wild-type viruses

EV71 and CA16 were isolated from a patient in Anhui (EV71-C4b) and Taiwan (CA16-B1a), respectively, during the HFMD epidemic in China, 2010. Both viruses were propagated in RDS cells. The culture supernatants were harvested 3 days post-infection, passed through a 0.45 μm filter to remove cellular debris and aliquotted for storage at −80°C as viral stocks.

#### EV71 and CA16 pseudoviruses

EV71 and CA16 pseudoviral vectors (CVA16-A: U05876.1, CVA16-B1a: AF177911.1, CVA16-B1b: EU262658.1, CVA16-B2: AY895127.1, EV71-B1: AB482183.1, EV71-B2: U22522, EV71-B3: AM396586, EV71-B4: AF316321, EV71-B5: EU527985, EV71-C1: DQ452074, EV71-C2: AF176044, EV71-C2L: HM622392.1, EV71-C3: DQ341356.1, EV71-C4a: AY895132.1, EV71-C4b: EU703814.1, EV71-C5: EF063152) (previously generated in our lab) were constructed by replacing the *P1* gene with a firefly luciferase reporter gene in the genome and inserting a CMV promoter at the 5′-end for transcription *in vitro*. 293T cells were transfected with the pcDNA3.1-T7 RNA polymerase expression plasmid, pT7-EV71/CA16-luc replicon plasmid and EV71 capsid expression plasmid using Lipofectamine 2000 (Invitrogen). EV71/CA16-pseudoviruses were collected 48 h post-transfection by two rounds of freeze-thaw cycles and then aliquot and stored at −80°C as viral stocks.

#### EV71-EGFP and CA16-EGFP viruses

Replication competent EV71 and CA16 with the *EGFP* reporter gene, EV71-EGFP and CA16-EGFP virus (respectively derived from EV71-C3, a gift kindly provided by Liguo Zhang at the Institute of Biophysics, Chinese Academy of Sciences, and from CA16-B1a, previously generated in our lab), were generated in 293S and RDS cells. The coding sequence of EGFP was inserted between the 5′UTR and VP4. 293T cells were transfected with EV71-EGFP and CA16-EGFP vectors using Lipofectamine 2000. Viruses propagated in 293S and RDS were collected after two rounds of freeze-thaw cycles, passed through a 0.45 μm filter to remove cellular debris, aliquotted and stored at −80°C as viral stocks.

### Establishment of cell lines

#### Lentivirus production from Lenti-X vector

The coding sequence of the human *SCARB2* gene was inserted into the Lenti-X pLVX-Puro vector (Clontech) with *EcoR* I and *Xba* I sites. For production of the lentivirus particles, 293T cells were transfected with the plasmid following the manual. The cell culture supernatant was harvested 72 h after transfection, passed through a 0.45 μm filter and centrifuged in 4000 × *g* for 5 min to remove cellular debris. Lentivirus production was detected in the supernatant using an anti-p24 monoclonal antibody (data not shown) before storage at −80°C.

#### Establishment of stable transgenic cell lines

The three parental cell lines (293, RD, Vero cells) were infected with the lentivirus carrying the *SCARB2* transgene. After 48 h of incubation, cells were passaged three times with 10% FBS-DMEM containing puromycin at a pre-determined dosage. The positively screened cell lines were sub-cloned three times by limiting dilution, and gene expression was detected every fifth passage via real-time RT-PCR and flow cytometry.

### Analysis of stable transgenic cell lines

#### Real-time RT-PCR

To detect the relative *SCARB2* expression at the gene level, mRNA was extracted from the six cell lines with the QIAamp RNeasy MiniKit separately according to the manufacturer’s instructions (QIAGEN). Reverse transcription was performed with TAKARA Reverse Transcriptase XL. PCR was carried out using the following primers: SF: 5′-GTACTGAGGCATTTGACTCCT-3′, SR: 5′-AGTTCCCTGTAGGTGTATGGC-3′. The real-time RT-PCR cycling program involved an initial denaturation step at 95°C for 2 min, followed by 40 cycles of 15 s at 95°C and 30 s at 60°C. Fluorescence data were collected during each annealing-extension step, and the relative *SCARB2* mRNA levels were analyzed with *β-actin* as the internal control using the Bio-Rad CFX96 software.

#### Western blot

Expression levels of SCARB2 in the positive cell lines were assessed by Western blot with tubulin as the internal control. After treatment with lysis buffer, cell lysates were subjected to SDS-polyacrylamide gel electrophoresis (SDS-PAGE). Following electrophoresis, the resolved proteins were transferred onto a nitrocellulose membrane and blocked with 5% evaporated milk for 15 min at 37°C. The membrane was then incubated with an anti-SCARB2 antibody (diluted 1:1000, Abnova: PAB13673) for 45 min at room temperature, followed by incubation with an alkaline phosphatase (AP)-conjugated rabbit anti-goat secondary antibody at a dilution of 1:1000. After 30 min of incubation at room temperature, staining was carried out with NBT and BCIP solutions.

#### Flow cytometry

Cells were harvested using trypsin-EDTA, which was neutralized with FBS-containing culture medium. The cells were pelleted and incubated with the SCARB2-specific antibody (diluted 1:100, Abnova: H00000950-M01) in PBS containing 1% BSA at room temperature for 30 min, followed by incubation with FITC-conjugated goat anti-mouse IgG. SCARB2 protein on the cell surface was detected on a Beckman flow cytometer. The fluorescence intensity was determined after deducting the background staining with the secondary antibody alone.

#### Localization of SCARB2

Cells which are susceptible to infection often express virus receptor proteins at the cell membrane. We detected the localization of SCARB2 using laser confocal microscopy. Generally, 293S, RDS and VeroS cells were cultured on microscope slides for 24 h before fixation with 4% paraformaldehyde for 10 min at room temperature. For each transgenic cell line, one slide was permeabilized with 0.25% Triton-100 for 8 min at room temperature, while another slide was not treated. After exposure of the slides to the primary anti-SCARB2 antibody overnight at 4°C, a FITC-conjugated goat anti-mouse antibody was used as the secondary antibody (diluted 1:100) to stain cells at room temperature for 45 min in a darkroom. Subsequently, the microscope slides were mounted with coverslips and 50% glycerol. Images were acquired using a fluorescence microscope.

### Characterization of stable transgenic cell lines

#### EV71 and CA16 pseudovirus infection

To compare the susceptibility to virus infection of the stable transgenic cell lines with that of the original cells, EV71 and CA16 pseudoviruses containing a luciferase reporter gene were used to infect the six cell lines. Cells (10^4^/well) were seeded in 96-well plates for approximately 12 h before infection. Various EV71 and CA16 pseudoviruses were applied to cells at 200 cell culture 50% infectious doses (CCID_50_)/well. The firefly luciferase activity was measured after 24 h of incubation using Thermo Fluoroskan Ascent FL [32].

#### EV71-EGFP and CA16-EGFP virus infection

Replication-competent EV71 and CA16 with an *EGFP* reporter gene were generated with the C3 and B1a infectious clones, respectively, by transfection of 293T cells using Lipofectamine 2000. EV71-EGFP and CA16-EGFP viruses were collected 48 h later and immediately co-cultured with 293S and RDS cell lines. The six cells (10^6^/well) were seeded in 6-well plates approximately 12 h before infection, and then viruses were added to the wells at the multiplicity of infection (MOI) of 0.01. After incubation for 48 h, images were acquired using a fluorescence microscope.

#### Replication kinetics

To examine viral growth kinetics, we infected the six cell lines (three transgenic and three parental cell lines) with wild-type virus (EV71-C4b or CA16-B1a) at an MOI ≈ 0.001. After incubation for 12, 24, 48 or 72 h, the viruses were harvested separately and frozen at −80°C until use. Thereafter, RD cells (10^4^/well) were seeded in a 96-well plate, and the viruses collected above were serially diluted 10-fold in DMEM with 2% FBS and added to the wells in 10 parallel sets. After incubation for 72 h, the CCID_50_ was calculated via the Reed-Muench method by determining the number of wells with CPE. The viral kinetics was formulated using the CCID_50_ of the six cell lines.

To examine EV71 and CA16 viral growth kinetics in the gene level, viruses collected at the four time points from the six cell lines were measured using real-time RT-PCR [39]. The viruses were purified prior to the real-time RT-PCR analysis. Briefly, the infected cells were lysed by three rounds of freezing and thawing. The resultant lysates were filtered through a 0.22-μm filter. Viruses were then purified from the resultant suspension by centrifugation at 28,000 rpm for 4 h in a Beckman SW40 rotor. Thereafter, the viral mRNA was extracted from those purified virus stocks with the QIAamp Viral RNA Minikit separately according to the manufacturer’s instructions (QIAGEN), and reverse transcription was performed with TAKARA Reverse Transcriptase XL. PCR to detect EV71 and CA16 was carried out using the following primers: VF (5′UTR: 445–459 bp): 5′-TCCTCCGGCCCCTGA-3′, VR (5′UTR: 580–600 bp): 5′-AATTGTCACCATAAGCAGCCA-3′. The cycling program involved an initial denaturation step at 95°C for 2 min, followed by 40 cycles of 15 s at 95°C and 30 s at 60°C. Fluorescence data were collected during each annealing-extension step and analyzed using the Bio-Rad CFX96 software.

## Competing interests

The authors declare that they have no competing interests.

## Authors’ contributions

XL and PF organized the study, took part in all experiments and drafted the manuscript. WK, CJ and FG designed the experiments and edited the manuscript. LX and WS, DA, JJ, SS, YZ and XM participated in production of EV71 and CA16 viruses and pseudoviruses. All authors read and approved the final manuscript.
